# Tranexamic acid therapy for postoperative bleeding after bariatric surgery

**DOI:** 10.1186/s40608-018-0213-5

**Published:** 2018-12-03

**Authors:** R. A. Klaassen, C. A. Selles, J. W. van den Berg, M. M. Poelman, E. van der Harst

**Affiliations:** 1 Department of Surgery, Maasstad Hospital, Maasstadweg 21, 3079 DZ Rotterdam, The Netherlands; 2 Department of Surgery, Franciscus Gasthuis & Vlietland, Kleiweg 500, 3045 PM Rotterdam, The Netherlands

**Keywords:** Tranexamic acid, Gastric bypass, Hemorrhage, Complications

## Abstract

**Background:**

Tranexamic acid reduces blood loss associated with various surgical procedures. Postoperative bleeding caused by dissection or bleeding of the enteric staple lines is a well-known complication following bariatric surgery. Reoperation in order to restore hemostasis is frequently necessary (up to 2.5% in literature). The effect of conservative therapy using tranexamic acid for postoperative hemorrhage after bariatric surgery is still very much a novel technique. The aim is to present our results (reoperation rate and thrombo-embolic complication rate) of tranexamic acid therapy for postoperative bleeding after bariatric surgery in comparison to those in existing literature.

**Methods:**

We retrospectively reviewed 1388 patients who underwent bariatric surgery (laparoscopic gastric bypass or laparoscopic gastric sleeve). Use of tranexamic acid, reoperation rate, transfusion rate and rate of thrombo-embolic complications were reviewed.

**Results:**

Forty-five of 1388 (3.2%) total patients experienced significant hemorrhage after bariatric surgery. Tranexamic acid was administered in 44 of these patients. A failure of the treatment with tranexamic acid was observed in four patients. The incidence of reoperation was 0.4% for the entire population. No thrombo-embolic complications were registered for patients receiving tranexamic acid.

**Conclusion:**

These findings suggest that the administration of tranexamic acid appears to be safe in reducing the reoperation rate for bleeding after bariatric surgery.

## Background

Obesity rates have been rapidly increasing worldwide the last decades. Bariatric surgery has been established as the standard treatment for morbid obesity. Bariatric surgery has been shown to be a safe treatment for the reduction of weight and associated comorbidities [1]. However, common postoperative complications following bariatric surgery include anastomotic leakage, intestinal obstruction, thrombo-embolic event and postoperative hemorrhage. The incidence of post-operative hemorrhage ranges from 0.5 to 4% [2]. These hemorrhages occur at staple lines, mesenteric vessels or caused by iatrogenic injury.

In larger series, reoperations to reach hemostasis have been reported in 0.8% to 2.5% of patients with postoperative abdominal hemorrhage following bariatric surgery [3]. To prevent reoperation caused by staple line bleeding, various techniques such as the use of fibrin sealants and the use of reinforcement strips have been tried [4, 5]. However, randomized data on the effect of these techniques in bariatric surgery do not exist. Reoperation remains the method of choice when conservative management fails in hemodynamically unstable patients.

Tranexamic acid (TA), a synthetic derivative of the amino acid lysine, is a potent antifibrinolytic drug. It prevents the binding of plasminogen to the surface of fibrin and reduces the activation of fibrin, resulting in inhibition of fibrinolysis [6]. Due to these recognized properties, there has been an increased interest in the usage of tranexamic acid in reducing bleeding. Several studies showed the positive effects of tranexamic acid to reduce peri-operative hemorrhage [7–10]. Administration of tranexamic acid has proven to reduce surgical bleeding in cardiac surgery, orthopedic surgery, and liver transplant surgery [11, 12]. Administration of tranexamic acid has also proven to have a significant effect in trauma patients [13].

The use of tranexamic acid against staple line bleeding in sleeve gastrectomy has been studied [14, 15]. In our hospital, over 500 bariatric procedures are performed annually. Since successful reports of tranexamic acid treatment in other surgical fields, it is commonly used as treatment for post-operative hemorrhage after bariatric surgery in our hospital.

The aim of this study was to evaluate our re-operation rate and thrombo-embolic complication rate with the use of tranexamic acid administration in cases of post-operative hemorrhage after bariatric surgery.

## Methods

Collection of data was performed with the approval of the Institutional Review Board. The medical records of patients who underwent bariatric surgery from August 2011 until December 2014 were retrospectively reviewed. Before this period our bariatric unit was still developing into the unit in which yearly approximately 800 patients undergo bariatric surgery. Patients with (suspected) significant post-operative bleeding complications were reviewed. Patients receiving tranexamic acid after bariatric surgery or patients re-operated due to postoperative hemorrhage after bariatric surgery were included. Patients receiving tranexamic acid as regular medication were excluded (*n* = 1).

Bariatric procedures in our center are laparoscopic gastric bypass, and laparoscopic gastric sleeve. The same stapler was used in all cases and no reinforcement was used. Wash is not used during bariatric procedures. During all procedures the surgeon routinely places an abdominal drain. The drain is usually removed one day postoperatively.

Postoperative hemorrhage was suspected in patients with one or more the following signs: postoperative tachycardia (> 100 bpm), hypotension (systolic pressure < 100 mmHg), an increased drain production (> 100 ml/24 h) or a drop in hemoglobin level of more than 2 mmol/l. In our institution it is common practice that in patients where the clinical diagnosis of suspected hemorrhage is high, a diagnostic laparoscopy is performed. This is due to the fact that suspected hemorrhage is not always visible on imaging. All patients received low molecular weight heparin (LMWH) 5000 IE postoperatively starting the following morning. The doses of tranexamic acid and hospital stay in patients where LMWH was discontinued were therefore compared with patients in whom administration of LMWH was continued. LMWH was not continued when the patient was discharged from the hospital.

Outcome measures were reoperation rate, transfusion rate and thrombo-embolic events with a follow-up of six months. Patients are seen for clinical follow-up every three months during the first year following bariatric surgery.

Patients who received tranexamic acid were treated with a dosage of 1000 mg in 100 cc of NaCl intravenously four times daily. This was given until the patient was deemed stable. Blood transfusion was given according to the Dutch Blood Transfusion Guideline [16].

### Data analysis

The percentages/incidence of re-operations, blood transfusions, and thromboembolic complications were compared with reported incidences. Normally distributed continuous variables were reported as mean and standard deviation, and compared using ample t-tests, and non-normally distributed continuous variables were reported as median and interquartile rage [IQR], and compared using two-sided Wilcoxon rank- sum tests. *P* values < 0.05 were considered statistically significant. Statistical analysis was performed with SPPS software (version 21, IBM).

## Results

### Patient selection

A total of 1388 bariatric procedures were reviewed from 2011 through 2014. 46 patients experienced hemorrhagic complications. Of these 46 patients, one was excluded due to the need of tranexamic acid as regular medication for von Willebrand disease, leaving a final study population of 45 patients. Baseline characteristics are shown in Table 1.

**Table 1:** Baseline characteristics

Variable	Hemorrhagic complications (*N* = 45)
Age (years), mean ± SD	42.6 ± 9.8
Female, n (%)	31 (69)
Surgical procedure	
LRYGB	35/1134
LRYGB after lap band	7/134
Gastric sleeve	2/85
LRYGB after sleeve	1/35
Hospital stay (days), median [IQR]	4 [3–5]
Operation duration (minutes), mean	81 ± 25
Comorbidity	
Hypertension	12
Diabetes	9
Asthma or OSAS	6
Hypercholesterolemia	3
Hypothyroidism	3
None	2

### Reoperation due to hemorrhage

In total, tranexamic acid was administered to 44 of the 45 patients who suffered significant postoperative bleeding. The incidence of reoperation due to hemorrhage was 0.4% (Table 2). Two patients were re-operated the same day as the primary surgery because of rapidly increasing hemodynamic-instability. One of these patients did not receive tranexamic acid because they were reoperated immediately. The other three patients were reoperated on day 1 (*n* = 2) and day 2 (*n* = 1). This results in a failure of the tranexamic acid treatment in four patients (9%). The origin of hemorrhage during re-operation was found to be the staple-line (n = 2), trocar opening (n = 1) and of diffuse origin (n = 2). Vital signs and intra-operative findings of these patients are demonstrated in Table 3. Patients who were reoperated had a median hospital stay of 5 [IQR 3.5–44.5] days compared to 3.5 [IQR 3–5] in patients who were not reoperated. There was no mortality in this series.

**Table 2:** Reoperation rate

	Total group (*N* = 1388)
Hemorrhagic complications	45 (4.0)
Reoperation	5 (0.4)
After receiving tranexamic acid	4
Immediate, no tranexamic acid	1

Presented as N, (%)

**Table 3:** Vital signs and intra-operative findings of patients who were reoperated

Patient	Re-operation day	Blood pressure (mmHG)	Pulse (bpm)	Hemoglobin (mmol/l)	Drain production (cc)	Imaging findings	Intra-operative findings
1	1	120/64	128	6.9	400	CT-abdomen: Fluid around pouch	Hematoma, 400 cc blood evacuated, no active bleeding, anastomoses intact
2	0	i) 93/55	90	–	–	–	Bleeding from trocar opening, 1000 cc blood evacuated, anastomoses intact
	4	ii) 107/60	115	6.0	–	X-thorax: free air CT-abdomen: Free fluid	Bleeding of gastro-jejunostomy anastomosis
3	1	127/81	104	5.7	650	–	Bleeding vasa brevia Bleeding anastomosis pouch
4	2	147/100	125	6.0	100	–	Liver laceration Bleeding spleen
5	0	74/55	80	6.3	0	–	Diffuse bleeding of staple line, vasa brevia

### Transfusion rate

The overall transfusion requirement was 19 of 1388 patients (1.4%). In the study population, 19 of the 45 patients received transfusions (42.2%). Four of the five patients reoperated received transfusions. Patients received one to four packed cells.

### Tranexamic acid

The median amount of five doses of tranexamic acid was administered (range 0–17). All patients received heparin postoperatively. However, in 29 (64.4%) patients LMWH was continued despite of the occurrence of a hemorrhage (Table 4). These patients did not require more tranexamic acid than those in which the heparin had been stopped (*p* = 0.52). In 30 cases (66.7%), tranexamic acid was administered on day 1 postoperative (median day 1.2, range 0–3). At this moment, the patient experienced signs of hypovolemia such as tachycardia, an increased drain production, and a decrease in hemoglobin level.

**Table 4:** LMWH continued vs. LMWH discontinued

	LMWH continued	LMWH discontinued	
Number	16	29	
Doses tranexamic acid (median)	5	5.5	*p* = 0.66
Hospital duration	3	6	*p* < 0.001
Reoperation rate (%)	25	3.4	*p* < 0.001

### Complications

No thromboembolic complications such as deep venous thrombosis or lung embolism were found in these patients at 6 months follow-up. No cases of acute kidney injury were found in the patients receiving TA.

## Discussion

There is ample research available on the effects of tranexamic acid in various fields of surgery such as trauma surgery, orthopedic surgery, cardiac surgery and liver transplantation surgery. However, little is known about the effect of tranexamic acid in bariatric surgery. Presumably clinicians are reluctant to use this medicine in fear of causing thromboembolic complications and rather reoperate to reach hemostasis. Chakravarrty et al., studied the effect of intraoperative tranexamic acid on staple line bleeding, and found that intraoperative prophylactic tranexamic acid use effectively reduces staple line bleeding [14]. Another study describes their positive experience with tranexamic acid use for reducing staple line bleeding [15].

In larger series an overall hemorrhage rate of 0.5–4% has been noted after bariatric surgery. The reported reoperation rate ranges from 0.8 to 2.5% compared to the relatively low incidence of 0.4% in our study. The transfusion rate ranges from 0.3 to 1.5% in the literature compared to 1.4% in our study [2]. Importantly we did not encounter any thromboembolic events up until six month postoperatively.

Clinical trials have evaluated the safety and efficacy of tranexamic acid in upper gastrointestinal bleeding [17]. In patients with upper GI bleeding tranexamic acid administration significantly reduces bleeding rate from 30 to 20%. The need for surgery in patients decreases from 40 to 30% [10]. Therefore, the use of tranexamic acid in surgery is expanding rapidly. In trauma patients the CRASH trial assessed the effects of early administration of tranexamic acid on death, vascular occlusive events and the administration of blood transfusion. All-cause mortality, as well as the risk of death due to bleeding was significantly reduced when tranexamic acid was administered. The CRASH 2 trial quotes a 1.7% incidence of vaso-occlusive events in the tranexamic acid group and 0.3% deaths due to vascular occlusion. This risk is compounded to up to 3% risk of thromboembolism in bariatric patients undergoing surgery. It should be noted that laparoscopic procedure in itself increases this risk [13]. Perioperative tranexamic acid in patients undergoing total knee arthroplasty resulted in a significant reduction in mean blood loss compared to placebo (940 mL and 1293 mL) [9]. During liver transplantation, intravenous infusion of tranexamic acid resulted in clinical benefits without episodes of arterial or venous thrombosis [18]. In spine surgery tranexamic acid reduced intraoperative, postoperative, and total blood loss of 219 mL, 119 mL, and 202 mL respectively. In addition tranexamic acid reduced the number of patients who received a blood transfusion [7].

In the latest reviews and meta-analyses, no increased risk of thromboembolic complications due to the administration of tranexamic acid has been found [11]. Despite these recent studies, concerns remain regarding the risk of thromboembolic events, especially in high-risk patient for postoperative thromboembolic events with higher systemic concentrations and prolonged intravenous application [19]. This may well be the reason why TA after bariatric surgery has not become a popular hemostatic tool.

Patients undergoing bariatric surgery commonly receive low molecular weight heparin as a prophylaxis for thrombo-embolic complications. Because the LMWH was given in the morning, patients with suspected hemorrhage had already had this dose administered before treatment of the suspected hemorrhage has taken place. However, LMWH was not always ceased in patients when hemorrhage occurred and tranexamic acid was administered. No significant difference was found in doses of tranexamic acid administered in patients where this was continued of discontinued. Hospital stay was twice as long for patients in whom LMWH was discontinued. Reoperation rate on the other hand was significantly lower for this group in which LMWH was discontinued. There is no sufficient explanation for these results. It appears that when patients are not reoperated and LMWH has been continued they are observed for a longer period. Since LMWH and TA counteract each other, we emphasize the importance of discontinuing LMWH when a patient is suspected of hemorrhage.

Postoperative hemorrhage in bariatric surgery is an uncommon complication. However, it remains the most common cause for reoperation within a 30-day period [3]. Adaptations in surgical techniques as well as pharmacological treatments may provide benefits in even more reducing this complication. New stapler techniques have been efficient in reducing the incidence of anastomotic leakage and therefore the reoperation rate. Various technical modifications such as the application of hemostatic agents on staple lines have been described to decrease the incidence of postoperative bleeding [20]. These developments have not been analyzed in relation to the prevention of reoperation.

Our study has a number of limitations, including its retrospective nature and the fact that it was conducted at a single institution. Our results, like those earlier published, demonstrate our experience with the use of tranexamic acid [15]. These results do not however demonstrate statistical proof of the effectiveness of tranexamic usage. It has become common practice in our center to administer tranexamic acid to patients with suspected hemorrhage after bariatric surgery, but reasons for administering tranexamic acid were not always clearly documented. An ideal study design for future research would be a randomized controlled multi-center trial. However, with an incidence of 0.4% in over 3 years, the numbers are extremely small to perform a randomized controlled trial.

**Fig. 1:**
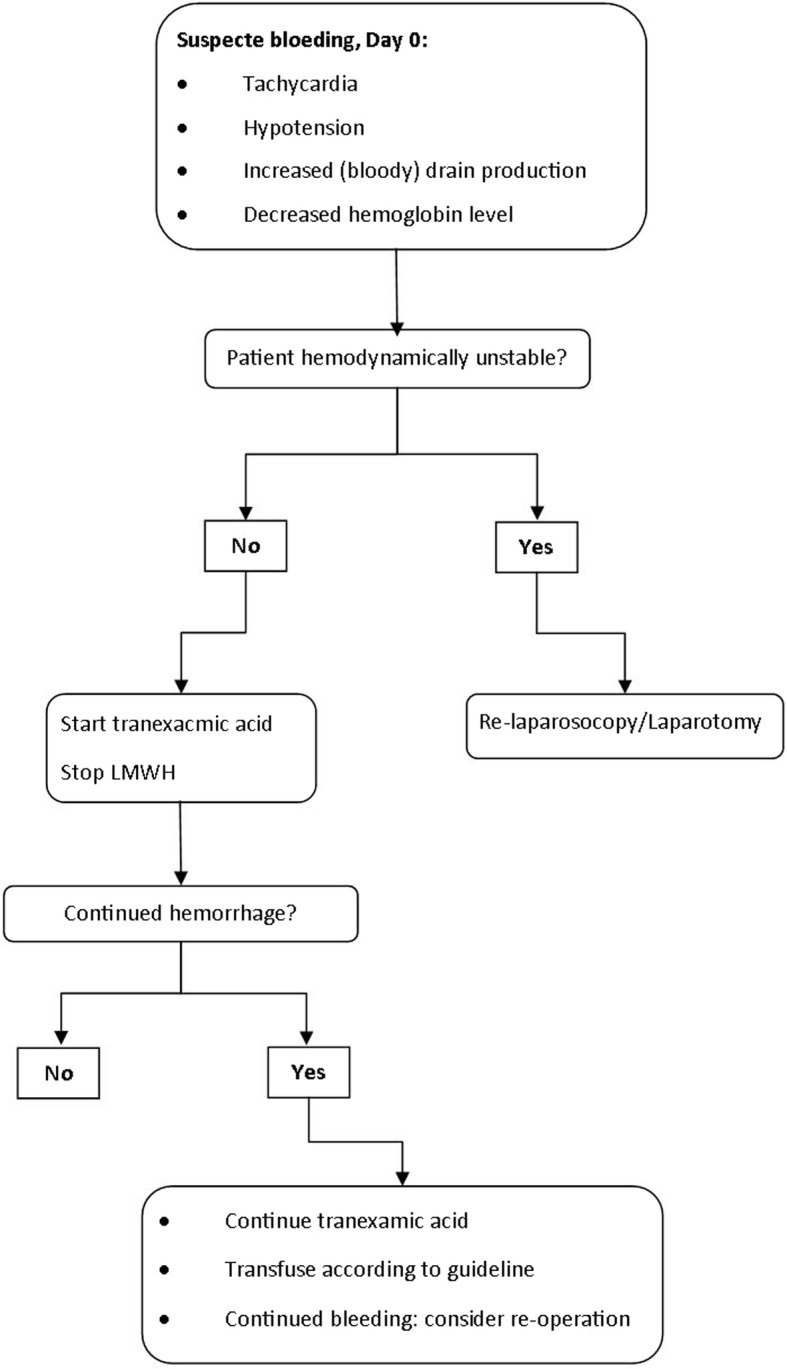
Proposed treatment algorithm

A treatment algorithm for the management of patients with postoperative hemorrhage after bariatric surgery is proposed in Fig. 1. Important parameters to take into account when evaluating the patient are: heart rates, blood pressure, drain production, and hemoglobin levels. Patients who are hemodynamically unstable should undergo a diagnostic laparoscopic reoperation. However, in the patients with suspected hemorrhage who are not in shock, this conservative approach with the use of tranexamic acid could be a treatment option.

## Conclusions

Tranexamic acid administration may be effective in reducing the reoperation rate for bleeding complications after bariatric surgery. In this study, tranexamic acid was not associated with increased thromboembolic events. Larger studies are necessary to confirm the safety and efficacy of this treatment approach.

## Abbreviations

IQR: Interquartile range
LMWH: Low molecular weight heparin
TA: Tranexamic acid
